# People’s explanatory preferences for scientific phenomena

**DOI:** 10.1186/s41235-018-0135-2

**Published:** 2018-11-21

**Authors:** Deena Skolnick Weisberg, Emily J. Hopkins, Jordan C. V. Taylor

**Affiliations:** 1 Villanova University, 800 E. Lancaster Ave., Villanova, PA 19085 USA; 2 University of Scranton, 800 Linden Street, Scranton, PA 18510 USA; 3 University of Pennsylvania, 3808 Walnut St, Philadelphia, PA 19104 USA

## Abstract

Previous work has found that people are drawn to explanations of psychological phenomena when these explanations contain neuroscience information, even when that information is irrelevant. This preference may be due to a general preference for reductive explanations; however, prior work has not investigated whether people indeed prefer such explanations or whether this preference varies by scientific discipline. The current study asked 82 participants to choose which methods would be most appropriate for investigating topics in six scientific fields. Participants generally preferred methods that either matched the field of investigation (e.g., biology for biology) or that came from the immediately more reductive field (e.g., chemistry for biology). Both of these patterns were especially evident for the pairing of psychology and neuroscience. Additionally, participants selected significantly more methods as being useful for explaining neuroscience phenomena. These results suggest that people’s sense of the relations among scientific fields are fairly well calibrated but display some general attraction to neuroscience.

## Significance

People tend to think that explanations of psychological phenomena are better when those explanations contain neuroscience information, even when that information is irrelevant to the logic of the explanation. One possible reason for why this might happen is that people have a general preference for explanations that include language from more fundamental fields (reductive explanations), and psychology can be seen as reducing to neuroscience. The study reported in this paper investigates whether this preference for reductive explanations is particularly strong for psychology, whether it leads participants to preferentially seek out neuroscience explanations, and whether this preference leads participants to be drawn to explanations from the immediately reductive level (e.g., biology for neuroscience) rather than from more reductive fields (e.g., physics for neuroscience). To do so, we asked participants to choose which methods would be most appropriate for investigating topics in six scientific fields. We found that participants generally preferred methods that either matched the field of investigation (e.g., biology for biology) or that came from the immediately more reductive field (e.g., chemistry for biology). However, people were somewhat more likely to reduce psychology to neuroscience and also selected significantly more methods as being useful for explaining neuroscience phenomena. These results have implications for how members of the public understand neuroscience information, especially in cases where dualist tendencies may encourage people to think about individuals as being separate from their brain processes (e.g., the law, clinical psychology, and psychiatry).

## Background

It is human nature to seek out explanations for phenomena in the natural world, and one of the main goals of the modern practice of science is to construct such explanations. A long tradition in philosophy has studied scientific explanations in general, proposing frameworks for deciding which explanations are true and what form such explanations should take (e.g., Garfinkel, 1981; Hempel & Oppenheim, 1948; Strevens, 2008). Explanations of psychological phenomena have posed a particularly interesting set of puzzles, as scholars have debated whether such explanations should fruitfully be made at the level of the mind or *reduced to* talk about the brain (van Riel & Van Gulick, 2016). In this article, we consider the question of whether people prefer reductive explanations for psychology and explore how people’s explanatory preferences may shape their understanding of science in general.

### Explanatory reduction

While explanations can take many forms, one common form is *reductive*: providing an explanation of a phenomenon in terms of smaller component parts or more fundamental processes (see Craver, 2007). Issues of reduction are not unique to psychology and neuroscience, as this explanatory form can be found across the sciences. Consider the relationship between chemistry and physics, for example. While it is logically possible that atoms (and other elements of a physical ontology) could exist without there being any molecules, it is not logically possible that molecules could exist without atoms. Atoms, then, are logically prior to molecules. If an explanation of a molecular phenomenon is translated in terms of atoms, and if the atomic translation does not omit any aspect of the molecular version of the explanation, then we can say that the explanation has been *reduced* from the chemical to the physical level (Kemeny & Oppenheim, 1956; Oppenheim & Putnam, 1958).

Similarly, in the case of psychology, brains can exist without our having to postulate minds. But if brains are responsible for executing mental processes, then minds cannot exist without brains. Brains are in this sense prior to minds, as atoms were prior to molecules in the previous example. Accordingly, neuroscientists often attempt to explain the neural processes that underlie behavioral, perceptual, and cognitive phenomena. A reductive neuroscientific explanation of a psychological phenomenon is successful if it adequately captures all the features of the phenomenon at the neural level of description.

In practice and in theory, reduction is easier between some sciences than others. Since molecules necessarily include atoms in their definitions, reducing a chemical unit (molecule) to a group of physical units (atoms) is relatively straightforward. But the ontological units of psychology are not so neatly identified. According to some theories, neural processes are not logically necessary constituents of conscious experiences or intentional behaviors. Further, psychological explanations often rely heavily on theoretical inference. Therefore, any attempt to reduce the psychological to the neural will itself rely upon theoretically informed, psycho-neural bridge laws (e.g., Nagel, 1961). To date, there is no scientific consensus regarding the structure of such laws, nor is there consensus regarding what counts as a scientific law generally, even within a discipline (Godfrey-Smith, 2008). This means that there is no generally agreed upon proper form for explanations of psychological phenomenon.

### The case of seductive allure

Regardless of the objectively correct form for explanations in psychology, these might not be the explanations that people subjectively find satisfying. Indeed, prior work has found that people’s subjective sense of understanding or satisfaction is often decoupled from whether or not an explanation is accurate (Trout, 2002, 2016). For example, people tend to prefer teleological explanations even when they are not appropriate, as for natural phenomena (Lombrozo & Carey, 2006). People also prefer longer explanations (Kikas, 2003; Langer, Blank, & Chanowitz, 1978), explanations with more causal factors (Zemla, Sloman, Bechlivanidis, & Lagnado, 2017), and explanations that additionally provide a vacuous label for a phenomenon (Giffin, Wilkenfeld, & Lombrozo, 2017).

One particular instance of this kind of error in explanatory preferences is known as the *seductive allure effect*: people without advanced training prefer explanations for psychological phenomena when those explanations include a neuroscience component, even when that component is irrelevant to the logic of the explanation. In the initial demonstration of this phenomenon, participants judged explanations for psychological phenomena that were either good or bad (i.e., circular) and that either did or did not contain irrelevant neuroscience jargon. This jargon was irrelevant primarily because it merely translated information already present in the description of the phenomenon into neuroscientific terms (e.g., replacing “how people process faces” with “how the fusiform face area in the brain responds to faces”); expert neuroscientists agreed that this added jargon did not explain why the phenomenon happened.

Participants tended to accurately judge the bad explanations as less satisfying than the good ones, but they also judged that explanations containing irrelevant neuroscience language were more satisfying, particularly the bad ones (Weisberg, Keil, Goodstein, Rawson, & Gray, 2008). Although neuroscience information is not seductive in all circumstances (Diekmann, König, & Alles, 2015; Scurich & Shniderman, 2014), this effect has been replicated both directly (Fernandez-Duque, Evans, Christian, & Hodges, 2015; Weisberg, Taylor, & Hopkins, 2015) and conceptually (Rhodes, Rodriguez, & Shah, 2014). Additionally, similar effects have been found in other domains. For example, bogus mathematical terms in paper abstracts about biology and social science phenomena led participants to rate these abstracts more highly (Eriksson, 2012).

Why does this effect happen? Several possibilities have been discussed in the literature, and these explanations generally fall into two categories: those that emphasize the unique properties of psychology or neuroscience and those that see this as an instance of more general issues in judging scientific explanations in all fields. In terms of the first category, the most prominent possibility is that people are naïve dualists, assuming that brain processes and mental processes are separate (e.g., Harris & Richert, 2008; Preston, Ritter, & Hepler, 2013; see reviews in Bloom, 2004; Musolino, 2015). Because neuroscience provides evidence that this assumption is false, neuroscience information becomes correspondingly more surprising and hence attractive. Other explanations in this category point to the fact that neuroscience uses expensive equipment (e.g., NIH’s $150 million BRAIN Initiative) and produces visually appealing images (McCabe & Castel, 2008). Yet another possibility along these lines is that psychology is not seen as scientific (Keil, Lockhart, & Schlegel, 2010; Lilienfeld, 2012), and so adding any other type of information to psychological explanations makes them seem better.

In terms of the second category, the seductive allure of neuroscience could be an instance of a more general preference for reductive explanations. The sciences can be seen as being organized in a reductive hierarchy, whereby social science (i.e., studies of the behavior of groups of people) could be reduced to psychology (i.e., studies of individuals’ behavior), which in turn could be reduced to neuroscience (i.e., the brain processes that underlie behavior), and so on through biology, chemistry, and physics (see Fanelli & Glänzel, 2013; Smith, Best, Stubbs, Johnston, & Archibald, 2000). On this explanation, people prefer neuroscience language in explanations of psychological phenomena not necessarily because of a fascination with neuroscience per se, but because they believe that reductive explanations are superior in general.

A recent study found empirical support for this latter explanation by presenting participants with scientific phenomena across six different fields (social science, psychology, neuroscience, biology, chemistry, and physics). As in the initial demonstration of the seductive allure effect, participants read one of four explanations for each phenomenon, crossing good or bad with the presence or absence of irrelevant language from the immediately more reductive field (e.g., an explanation for a biological phenomenon that either did or did not include irrelevant language from chemistry). Participants judged explanations that contained the irrelevant reductive language as better across the sciences (Hopkins, Weisberg, & Taylor, 2016). This finding suggests that the seductive allure of neuroscience is a more specific instance of a general preference for reductive explanations: a *reductive allure*.

However, the effect of bogus reductionist language was somewhat stronger for explanations that reduced psychology to neuroscience. This suggests that neuroscience additionally has some unique features that might play an independent role in people’s judgments. What is it about neuroscience that leads to this special attraction? Or, conversely, what is it about psychology that makes people more likely to prefer a reductionist explanation for these phenomena? The current study was designed primarily to answer these questions, using a different method than in previous work. Rather than asking participants to rate explanations, we presented participants with scientific phenomena and asked them to select which investigative methods (e.g., “analyzing neural activity”) they thought would be appropriate for a variety of scientific phenomena. The main impetus behind using this method was to provide participants with more freedom to tell us about how they see the inter-relations among sciences, serving as a proxy for their explanatory preferences. By providing only brief descriptions of methods without any specific content, this task avoids a potential criticism levied at earlier work: that the irrelevant reductionist information included in the explanations used in those studies may have genuinely improved these explanations in some way, even though it was verified by experts to be non-explanatory. This current task, by providing only abstract investigative methods, ensures that participants are choosing among exactly the same kind of information for every item.

Another advantage of the current method is that it allows us to investigate a wider range of questions than previous work, specifically the issue of how people view reduction across the entire scientific hierarchy. Hopkins et al. (2016) provided participants with explanations that included language from the immediately more reductive level (e.g., chemistry for a biology phenomenon), but that study did not investigate whether there was a general preference for ever-more reduction (e.g., physics for a biology phenomenon). After all, many arguments in science and philosophy emphasize that the ultimate source of all of these phenomena are physics, or at least that all scientific explanations will ultimately consist of physical terminology (e.g., Carnap, 1937; Neurath, 1931; see Huttemann & Papineau, 2005; Stoljar, 2010 for analyses). Do people agree that this is the right way to explain all of science; are people maximally reductive? Previous work also has not investigated whether people might prefer explanations at a higher level (e.g., social science for a psychology phenomenon). Are there cases where referring to the larger or higher-order structures provides a satisfying explanation for a particular phenomenon?

The current study thus aimed to investigate the issue of whether and how the relationship between psychology and neuroscience might be special in the context of the full reductive hierarchy of the sciences using a novel method to explore this question: asking participants to select which methods would be appropriate for investigating phenomena from five different sciences. This method allowed us to investigate four main hypotheses. First, based on findings from previous work, we predicted that people would be more likely to choose neuroscience as an investigative method for psychological phenomena than to choose reductive methods for any other science; neuroscience itself is alluring as well as being at the potentially privileged reductive level for psychology. Second, we predicted that participants would be less likely to choose methods from psychology than methods from any of the other sciences, given prior work showing a general skepticism about psychological research.

Third, we predicted that people would be more likely to choose neuroscience as an investigative method for social science phenomena. Even though neuroscience is two levels below the social sciences in the hierarchy, rather than just one, we predicted that people would be attracted to neuroscience methods whenever they were available as a reductive option. Fourth, and conversely, we predicted that participants would be less likely to choose any reductionist methods (chemistry or biology) when asked which methods should be used to investigate a neuroscience phenomenon.

Additionally, we aimed to investigate people’s general preference for reductive explanations, as found in Hopkins et al. (2016), using a method that allowed people to select any method as appropriate for investigating any phenomenon. We particularly wanted to see whether our participants would prefer methods from the field that was immediately below the phenomenon in the hierarchy or whether they might prefer maximally reductive explanations, choosing methods from physics for all phenomena.

## Methods

### Participants

We recruited a group of undergraduates from an introductory psychology participant pool (*N* = 40; 11 men, 29 women; mean age 19.5 years; age range 18–22 years) and a group of workers from Amazon’s Mechanical Turk (MTurk) (*N* = 42; 21 men, 20 women, 1 unreported; mean age 38.0 years; range 20–77 years). Undergraduates received course credit for their participation, and MTurk workers were paid $0.50. An additional nine undergraduates and four MTurk workers consented but were not included in the final analysis due to failing an attention check trial (see below).

This research was performed in accordance with the Declaration of Helsinki and was approved as exempt from ethics approval by the University of Pennsylvania Institutional Review Board. Informed consent was obtained from all participants prior to their engagement in the study.

### Materials

We used the same 20 descriptions of scientific phenomena and their accompanying questions as in Hopkins et al. (2016), four from each of five fields of science (chemistry, biology, neuroscience, psychology, and social science). However, it is important to note that no explanations were provided in this study. Participants read only the description of the phenomenon and the target question that this description raised.

For example, one of the descriptions read, “People’s word-association abilities change depending on how long it takes them to say the first word that comes to their mind. Words that are said very quickly after hearing the target word are likely to sound similar to the target. However, words that are said after a short delay are more likely to have a similar meaning to the target. *Why does timing affect the type of words people associate with a target?*” All of these items are available online at https://osf.io/qw4dg/ and in the Appendix.

We chose not to include the physics items from the earlier study because we specifically wanted to investigate the extent to which participants would be attracted to reductive methods of investigation, and physics is at the bottom of the reductive hierarchy.

### Procedure

After providing consent, participants read a randomly selected subset of the 20 phenomena, two from each of the scientific fields for a total of 10 phenomena per participant. Each phenomenon description was provided individually. Participants were asked to read the phenomenon description and its accompanying question, and then to select which of six methods could help scientists answer the research question: analyzing atomic structure, analyzing chemical composition, analyzing tissue samples,^1^ analyzing neural activity, analyzing human behavior, and analyzing society-level statistics. These methods were phrased to reflect a primary investigative strategy used by our six target sciences: physics, chemistry, biology, neuroscience, psychology, and social science. The six methods were presented in a pre-determined random order for all items.

In response to this first question, participants were able to choose as many methods as they liked. If participants selected more than one, they received a second probe, asking them to choose which of their selected methods would be the *best* way to answer the research question.

After responding to the first five phenomena, participants engaged in an attention check (based on methods described in Oppenheimer, Meyvis, & Davidenko, 2009). The layout of this trial was superficially similar to the others in that it presented a one-paragraph text description followed by an italicized research question and the same choice options as the other trials. However, the text of the paragraph told participants explicitly to select all six choices to demonstrate that they were paying attention. Nine undergraduates and four MTurk workers who did not do so were excluded from the analysis.

Participants also engaged in two additional measures after they completed their selections for all 10 of their assigned phenomena. We first asked participants to match the six methods we used as choices in this study to their respective fields. For each method, participants were asked to select the fields in which that method is likely to be used. They were told that each method may be used in more than one field, and each field may be selected for more than one method.

The second additional measure asked about participants’ perceptions of nine scientific disciplines: physics, chemistry, biology, neuroscience, psychology, anthropology, political science, economics, and social science. This measure was based on Fernandez-Duque et al. (2015) and was also used in Hopkins et al. (2016). Participants were asked to rate each discipline’s scientific rigor (which we described as how closely its practitioners adhere to the scientific method), the size of the knowledge gap between experts in the field and average people, and how socially prestigious this field is (noting that we were not interested in the participant’s personal beliefs, but rather in how society sees the field). Ratings for all three of these questions were made on a scale from 1 to 10.

At the end of the survey, participants provided basic demographic information. Undergraduates reported their gender, age, class year, and current or anticipated major. MTurk workers reported their gender, age, highest level of education completed, and field of highest degree. All participants also reported whether they had taken college- or graduate-level courses in physics, chemistry, biology, neuroscience, psychology, anthropology, political science, economics, sociology, or philosophy.

## Results

Preliminary analyses found no differences by education or gender for the number or type of methods selected. There were a few differences between the MTurk workers and the undergraduates, which we will note as they arise, but since these were rare and minor we collapsed across these two groups for our analyses. Finally, we removed from analysis one of the items from the biology set because it did not pattern with the rest of the biology items, possibly because it presented an organism-level behavior (lizard mating rituals) while the other three presented sub-organismal phenomena (e.g., the action of cancerous cells). We address this issue more fully in the Discussion.

Unless otherwise noted, all analyses were mixed-effects logistic regressions predicting whether participants selected the level of interest on any given trial from the science of the phenomenon, including random intercepts for each subject nested within the sample to account for the repeated measures. Science was deviation coded such that the coefficient for each represents the difference between trials presenting a phenomenon from that science and the grand mean of all trials.

### Primary analyses

**Fig. 1:**
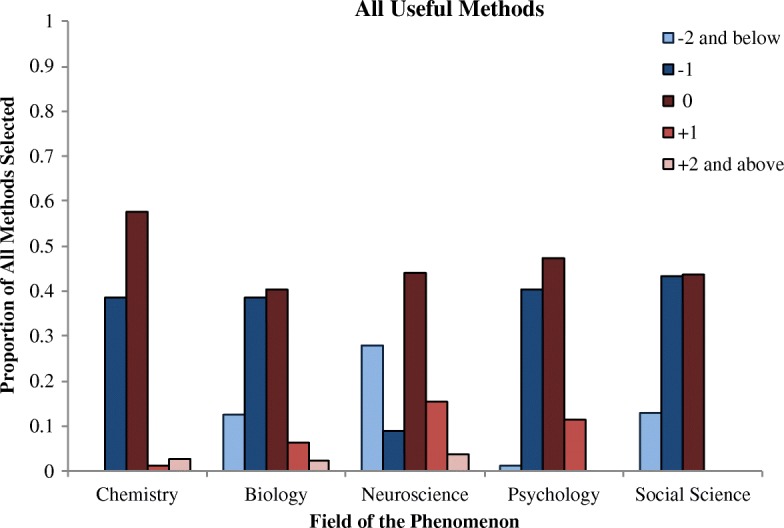
Proportion of times each level method was selected on the all useful methods question, broken down by the field of the phenomenon

**Fig. 2:**
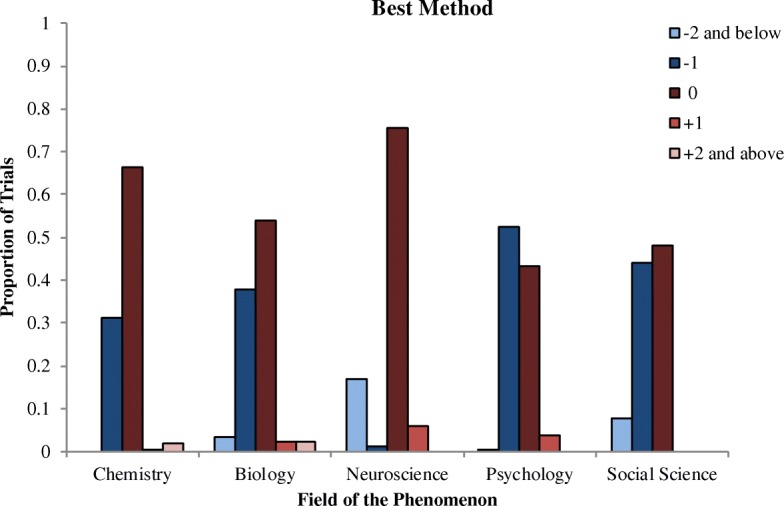
Proportion of times each level method was selected on the best method question, broken down by the field of the phenomenon

The main goal of this study was to confirm the uniqueness of the relationship between neuroscience and psychology as well as neuroscience’s privileged place in the hierarchy of the sciences. To do so, we coded each method for how many levels it was away from the field of the phenomenon (see Figs. 1 and 2). For example, on psychology phenomena trials, the social science method was coded as +1, the psychology method was coded as 0, the neuroscience method was coded as −1, the biology method as −2, the chemistry method was coded as −3, and the physics method was coded as −4.

We investigated four hypotheses. First, we predicted that participants would be more likely to choose the method at the immediately more reductive level for psychology (level −1; i.e., neuroscience) than for any other science. We thus compared how often this level was chosen both when participants were asked to select all useful methods and when they were asked to select the single best method. On the All Methods question, participants were significantly less likely than average to select the level −1 method on neuroscience trials (*B* = −1.54, SE = 0.16, *p* < .001); all other sciences were significantly above the average (all *p*’s < .05). However, neuroscience was a clear outlier here; the odds of picking the level −1 method on all trials (chosen on 33% of trials) were 5 times the odds of picking the level −1 method on neuroscience trials (chosen on 9% of trials). This large difference between neuroscience and the other sciences may have obscured any differences among the other sciences in this analysis. Thus, we repeated this regression with neuroscience trials removed. In this analysis, no sciences were significantly different from the average.

On the Best Method question, participants were again significantly less likely than average to select the level −1 method on neuroscience trials (*B* = −3.23, SE = 0.57, *p* < .001). The odds of picking the level −1 method on all trials (chosen on 33% of trials) were 40 times the odds of picking the level −1 method on neuroscience trials (chosen on 1% of trials), so again we ran the analysis with neuroscience trials removed. In this analysis, participants were significantly more likely than average to choose the level −1 method on psychology trials (*B* = 0.46, SE = 0.14, *p* < .001, odds ratio [OR] = 1.55). No other sciences were significantly different from the average. Our first prediction was thus partially supported: participants were more likely to pick the level −1 method on psychology trials when asked to pick the best method, but not when asked to pick all useful methods.

**Fig. 3:**
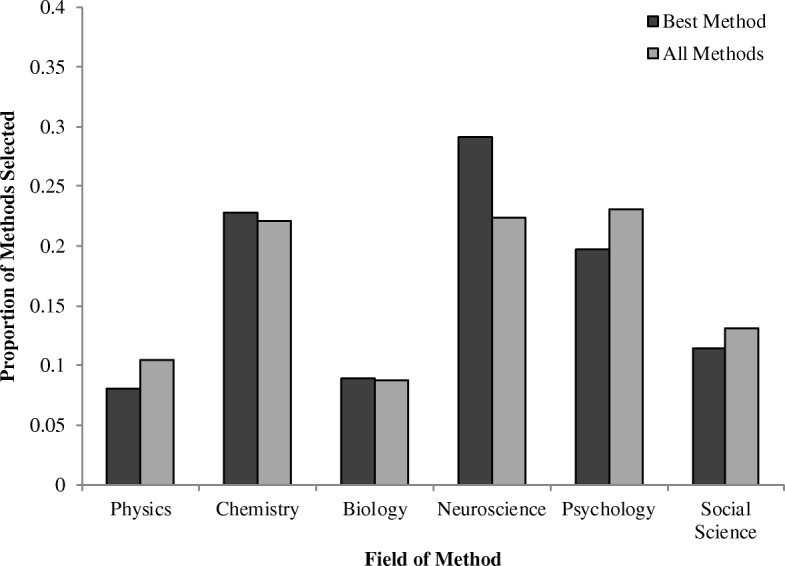
Proportion of times each method was selected across all phenomena

Second, we predicted that participants would be less likely to choose psychology as a method overall (Fig. 3). However, on the All Methods question, psychology was chosen more than any other method (23% of the time). On the Best Method question, the psychology method was chosen 20% of the time, behind neuroscience (30%) and chemistry (23%), but ahead of social science (11%), biology (9%), and physics (8%). Our data thus did not support this prediction.

Third, we predicted that participants would be more likely to choose the method at two levels down in the hierarchy for social science (level −2; i.e., neuroscience) than for any other science. On the All Methods question, we first removed the chemistry and psychology items because the level −2 method was never selected for either. For the remaining items (social science, neuroscience, and biology), participants were significantly more likely than average to choose the level −2 method on neuroscience trials (*B* = 0.46, SE = 0.13, *p* < .001, OR = 1.48). On the Best Method question, the level −2 method was selected too rarely (< 5% of all trials) to perform statistical analysis on differences between the sciences. This prediction was thus not supported.

Fourth, we predicted that participants would be more likely to choose the explanatory method at the same level (0) for neuroscience than for any other science. On the All Methods question, participants were more likely to choose the level 0 method for chemistry than for all sciences combined (OR = 1.56). When the chemistry trials were removed from the analysis, there were no significant differences between any of the other sciences. On the Best Method question, however, we found evidence to support our prediction: participants were significantly more likely than average to pick the level 0 method for neuroscience (*B* = 0.80, SE = 0.16, *p* < .001, OR = 2.27) compared with all trials combined. Interestingly, they were also significantly less likely than average to pick the level 0 method for social science (*B* = −0.40, SE = 0.14, *p* < .01, OR = 0.68) and psychology (*B* = −0.60, SE = 0.14, *p* < .001, OR = 0.56).

### Secondary analyses

We additionally wanted to discover whether participants would show a general preference for reductive explanations, as found in previous work. However, this was not the case in the current study. On the All Methods question, participants selected horizontal (46%) and reductive (45%) methods at roughly equal rates; methods from higher-level sciences were selected far less often (9%). When they were asked to select the best method, they chose horizontal methods (58%) significantly more often than reductive methods (39%; *p* < .001).^2^ Again, methods from higher-level sciences were selected rarely (3%).

We next tested to see whether participants tended to choose methods that were more reductive than level −1. Chemistry trials were excluded from this analysis because there was only one possible reductive method to choose, and the four trials where participants selected methods from level −4 or greater were not included. On average, participants selected the level −1 method on 54% of trials, the level −2 method on 21% of trials, and the level −3 method on 4% of trials on which it was an option. Participants were significantly more likely to choose the level −1 method than the level −2 method (*B* = 1.67, SE = 0.13, *p* < .001).^3^ They were also significantly more likely to choose the level −2 method than the level −3 method (*B* = 1.93, SE = 0.25, *p* < .001). A similar pattern was observed on the Best Method question: on average, participants selected the level −1 method on 31% of trials, the level −2 method on 6% of trials, and the level −3 method on 1% of trials on which it was an option (two trials where levels −4 and −5 were selected were not included). Participants were significantly more likely to choose the level −1 method than the level −2 method (*B* = 2.01, SE = 0.19, *p* < .001). They were also significantly more likely to choose the level −2 method than the level −3 method (*B* = 1.61, SE = 0.44, *p* < .001). Thus, participants tended to prefer methods from one to two levels below the field of the phenomenon, rather than being minimally or maximally reductive.

**Fig. 4:**
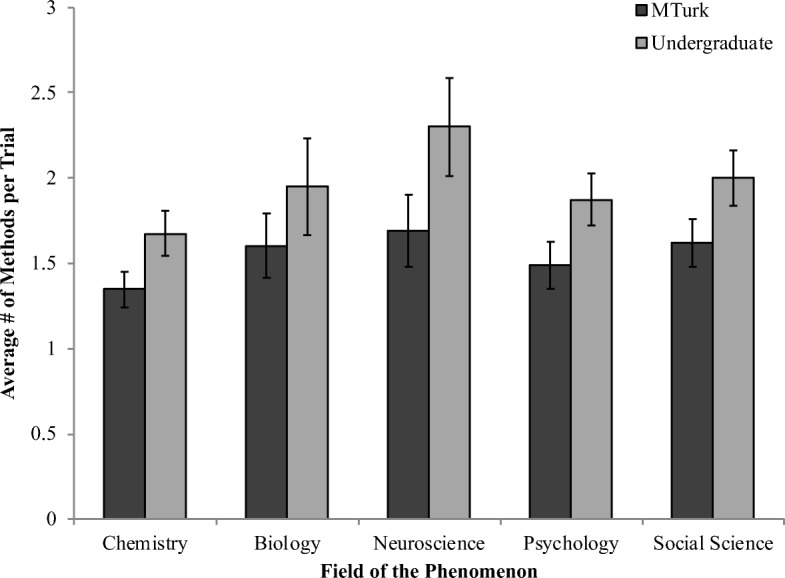
Average number of methods selected on the all useful methods question by sample and field of the phenomenon

Finally, we examined the total number of methods participants selected when asked to pick any methods they thought would be useful (Fig. 4). We conducted a mixed-effects regression predicting number of methods selected per trial from the sample and field of the phenomenon, controlling for random effects of participant. First, undergraduate students selected significantly more methods per trial than did MTurk workers (*B* = 0.42, *p* < .001). Collapsing across the samples, participants selected significantly fewer methods for chemistry compared with the sample overall (*B* = −0.25, *p* < .001), and significantly more methods for neuroscience (*B* = 0.23, *p* < .001).

### Auxiliary analyses

**Fig. 5:**
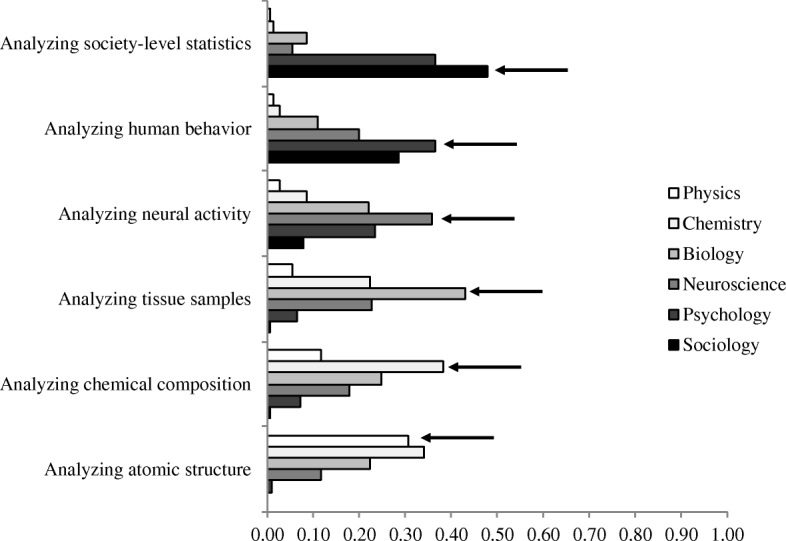
Responses when asked to match methods to fields. Arrows indicate the intended field for each method

An examination of participants’ responses to the additional measure asking them to match our descriptions of the methods to scientific fields revealed that they tended to agree that these descriptions belonged to the fields that we had intended. For each method except for “analyzing atomic structure”, the intended field was the most frequently selected (Fig. 5). This confirms that participants’ choices in the main task can be used to see their preferences for which fields would be most useful in answering the target questions, as we intended.

We also examined whether participants who think more highly of a particular field were more likely to select methods from that field in response to our main questions. To do so, we tested whether the number of times participants picked a method from a particular science correlated with their prestige rating of that science. However, we found no significant relations after removing outlier values and correcting for multiple comparisons.

## Discussion

People’s preferences for scientific explanations do not necessarily match the objective goodness of those explanations. The seductive allure effect—a preference for irrelevant neuroscience information in explanations of psychological phenomena—is one example of this disconnect, as people’s intuitive sense of satisfaction with an explanation seems to be based on the presence of neuroscience information and not on the objective quality of the explanation. Hopkins et al. (2016) demonstrated that this effect is an instance of a general preference for reductive explanations. However, that study was not able to determine the extent of this preference, since it only provided reductive explanations that drew from the immediately lower level.

To address this issue and to investigate further the factors that contribute to people’s intuitive sense of satisfaction, here we focused on the role of the explanatory level, asking participants to report which methods would be appropriate for investigating scientific phenomena from a variety of fields. In line with our expectations, we found that participants were more likely to choose neuroscience methods for psychology, although this trend was only evident on the question asking them to select the single best method and only after the neuroscience items were removed from the sample. This suggests that neuroscience may have some special status with respect to psychology, but that this is not a strong preference.

Further, and contrary to some work suggesting that people are generally hostile to the idea of psychology as a science, participants were not more likely to be reductionist for psychology than for the other sciences or avoidant of psychology methods as explanatory tools. This is particularly evident when looking at performance with the social science items, which participants generally preferred to be explained either in terms of social science itself or in terms of psychology. Participants thus do not generally seek to avoid the language or methods of psychology in scientific explanations.

Additionally, an analysis of participants’ overall preferences for reductive methods found that they generally preferred explanations that used methods of the same field as the phenomenon or the immediately more reductive field. Interestingly, this response tendency means that people are not maximally reductive, since physics methods were not generally seen as useful for explaining most scientific phenomena. These results are somewhat at odds with the findings from Hopkins et al. (2016), which found that people rated reductive explanations more highly than horizontal explanations. However, that study’s method was quite different from the current one. That study provided people with actual explanations and asked them to rate the quality of these explanations, whereas here we provided a choice of abstract investigative methods (rather than the text of explanations) and we asked participants to choose among all possible options (rather than having them rate the items one at a time). Given that the methods used by Hopkins et al. (2016) resemble more closely the circumstances under which people tend to encounter scientific explanations, we tend to believe that there is a general preference for reduction. However, the current study adds nuance to that finding by showing that reduction is not always seen as a virtue, and that people are not attracted to ever greater amounts of reduction in their explanations. These results thus indicate a reasonable degree of knowledge about how science works, indicating that people’s explanatory senses are not generally mis-calibrated with respect to scientific explanations.

However, there was one notable exception to these general trends: neuroscience. Specifically, participants were more likely to choose neuroscience methods for studying neuroscience questions than they were for choosing same-level methods for any of the other sciences, indicating that neuroscience is seen as having some kind of unique explanatory status. Additionally, participants tended to prefer a much higher number of methods to explain neuroscience phenomena than to explain phenomena from other fields. This means that participants see explanations for neuroscience phenomena as benefiting from input from a wider variety of sciences than average. Neuroscience is thus seen as being special among the sciences—not necessarily in the range of phenomena that it can be used to explain, but in the range of phenomena needed to explain it.

This response tendency may help to shed light on earlier findings about the seductive allure of neuroscience in explanations of psychological phenomena (Weisberg et al., 2008, 2015). In general, people seem to be attracted to neuroscience language, and the current study suggests that this might be because they see neuroscience as being more tightly connected to other sciences. By using neuroscience language in an explanation, people might think it is superior in having a broader basis (see Lombrozo, 2006).

### Limitations

One limitation of the current work is its use of a fairly restricted set of phenomena (four per discipline), which may not be representative of the full diversity of the sciences in question. Indeed, one of the items from the biology set patterned differently from the others, possibly because it described a behavioral phenomenon at the level of the organism that paralleled more closely some of the items from the psychology set than the other items in the biology set. Other sciences are similarly diverse, and phenomena drawn from different sub-fields of each domain may elicit different preferences, which we were not able to capture in this study and which may limit the generalizability of our results. The phenomena in the current study were the ones used in Hopkins et al. (2016), which allowed us to make direct comparisons between this study and that study, but future work should expand on the range of phenomena presented.

A related limitation is our choice of the methods associated with each domain. As noted above, our primary consideration in the current study was to ensure that each method was seen as representative of a single domain, so that our participants would be able to respond consistently to our test question. As with the issue of phenomena, this goal led us to exclude other methods at use in our target domains. Not all biologists analyze tissue samples, for example, and not all psychologists focus on human behavior. Again, future work should seek to add nuance to the presentation of scientific questions and methods from the different domains.

### Potential applications

These results have important implications for how people understand explanations of scientific phenomena in their everyday lives. Such explanations are ubiquitous, as people decide which medical treatments to seek out, for example, or how to make lifestyle choices that will reduce their carbon footprint. In general, most people’s understanding of mechanistic systems (e.g., bicycles) is much shallower than they believe, leading them to overestimate their knowledge in an *illusion of explanatory depth* (Lawson, 2006; Rozenblit & Keil, 2002). If people do not know what they do not know, they may not stop to question their initial judgment of an explanation. In many cases, as shown here, this may not be a problem—people’s understanding of what sort of information is relevant to answering a question seems to be generally intact. However, in the case of neuroscience, this intuitive sense of understanding might lead people astray. Specifically, given their tendency to see a larger number of sciences as relevant to neuroscience explanation, people might think that they need more information than they actually do to understand a neuroscientific phenomenon.

One particular arena where this tendency could become increasingly prominent is in law, where neuroscience has the potential to change everything (or perhaps nothing: Greene & Cohen, 2004). Some have argued that providing judges or juries with neuroscience information about a defendant (for example, that the defendant has atypical brain physiology or function) could sway legal decisions (Morse, 2011; but see Schweitzer et al., 2011). Indeed, some research finds that this is the case. For example, a study of judges found that they assigned significantly shorter sentences when they were provided with a biomechanism for a convict’s psychopathy than when they were not (Aspinwall, Brown, & Tabery, 2012). A similar study found that describing an event as caused by a physiological response, rather than in psychological terms, leads people to see the action as less voluntary (Monterosso, Royzman, & Schwartz, 2005), which could affect decisions about culpability.

A second area where these effects are likely to become evident is in clinical psychology. Previous work has shown that clinicians tend to see certain disorders (e.g., depression) as more psychological, and recommend therapy as a treatment for them, while they see other disorders (e.g., schizophrenia) as more biological, and recommend drugs as a treatment for these (Ahn, Proctor, & Flanagan, 2009; Miresco & Kirmayer, 2006). Disorders that are viewed as more biological are also viewed as being less under a patient’s control, but this can cut both ways: patients with the disorder may be seen as less responsible for their actions, and thereby less blameworthy, but they also may be viewed as more different from neurotypical people, and thereby less worthy of sympathy.

## Conclusions

At the heart of all of these decisions lies a series of assumptions about the interplay between mind and brain. Within academic fields, it is a live issue as to how to correctly understand this relationship, as noted in the Introduction, although there is general agreement that the mind and brain are not intrinsically separate (“the mind is what the brain does”). However, taken for granted in public discussions of these issues is the assumption of dualism: the mind and brain have different explanatory roles to play and may be separate entities (Preston et al., 2013). That is, members of the public—and even trained neuroscientists (Mudrik & Maoz, 2015)—operate under the assumption that the mind does not straightforwardly reduce to the brain. People may create even finer divisions, splitting the brain from both the mind and the soul (Harris & Richert, 2008).

This dualist assumption is likely to be inaccurate, but whether it is harmful is an open question. While it might lead to changes in sentencing practices, as noted above, it is unclear as to whether those changes are wrong in some objective sense. It could even be argued that the assumption of dualism could be a helpful metaphor or heuristic for understanding the vastly complex origins of our beliefs and behaviors. Regardless, this body of work makes clear that the issue of both actual and explanatory dualism is far from settled.

## Appendix

**Chemistry**

1. Wine is an alcoholic beverage made by pressing grapes and fermenting their juice. Wine is thus made up partially of alcohol, partially of water, and partially of dissolved grape solids. Some bottles of wine also contain small crystals, known as “wine diamonds,” which often can be seen sparkling on the bottom of the cork. Wine diamonds are more common when the wine is cold.
  *Why do wine diamonds form in cold wine?*
2. Usually, chewing food doesn’t have any effect other than to break the food down into smaller pieces. But crunching a piece of hard candy between your teeth may additionally release a spark of light for a brief moment. This effect is known as “triboluminescence.” It will happen with just about any type of hard candy, but it is particularly noticeable with wintergreen flavored mints.
  *Why is the triboluminescence effect stronger with wintergreen mints?*
3. Smog is a type of air pollution that makes breathing difficult and reduces how far one can see into the distance. In the 1990s, the city of Los Angeles had an especially bad problem with smog. Although smog was present nearly every day of the year, it appeared to be much denser and darker when the weather was hot.
  *Why does smog look denser when the weather is hot?*
4. Graphite and diamond are both made primarily of carbon. However, despite their nearly identical makeup, their properties are very different. Graphite is soft, dark gray or black, and is used as an industrial lubricant to make machine parts more slippery. Diamond is extremely hard, colorless, and can be used in blades and abrasives for cutting or grinding other materials.
  *Why does carbon sometimes form soft graphite rather than hard diamond?*

**Biology**

1. Cancer occurs when a buildup of cells in one part of the body forms a tumor that spreads to other tissues. Although some tumors are benign, malignant tumors can lead to a breakdown of normal bodily functions. Cancer cells can also break away from the original tumor and spread throughout the body, making treatment difficult.
  *Why do tumors form?*
2. The Hawaiian bobtail squid lives in a symbiotic relationship with a species of bacteria known as *Vibrio fischeri*. The bacteria benefit from the relationship because they live inside the squid and receive necessary nutrients from it. The squid also benefits from the relationship: the bacteria protect the squid by helping to hide it from predators while it hunts at night.
  *Why are the bacteria able to protect the squid from predators?*
3. The waves in the wake of a ship on the ocean at night will often appear to glow. The glow is not reflected light from the moon or the lights of the ship. Rather, it is produced by organisms in the ocean. The process by which some organisms produce their own light is known as “bioluminescence.”
  *Why do these bioluminescent organisms produce light?*
4. Male anole lizards bob their heads up and down rhythmically as part of a mating ritual to attract females. They typically increase their rate of head-bobbing when they see a female lizard of their species. However, their rate of head-bobbing also increases when they see another male lizard of the same species, even if no female lizards are present.
  *Why do male lizards bob their heads when other males are nearby?*

**Neuroscience**

1. Neuroscience research uses numerous techniques to observe the human brain. In order to gather important data about brain activity, such as a cognitive defect caused by a stroke, different brain scanning techniques can be employed. Functional magnetic resonance imaging (fMRI) is a crucial tool in the study of neuroscience. It allows for the measurement of patterns of activation by monitoring blood flow in an active brain.
  *Why does fMRI use blood flow as an indicator of brain activity?*
2. Some pharmaceutical drugs are designed to treat depression by affecting levels of serotonin, a chemical used by neurons in the brain for communication. When released from one neuron, serotonin binds with receptors on another and activates it. Lower levels of serotonin in the brain leads to less activation of these neurons. One class of antidepressants work by increasing the brain’s serotonin activity.
  *Why are serotonin-based treatments effective at combatting depression?*
3. Exposure to artificial light may be disruptive to sleep. This is especially true for blue light from high efficiency light bulbs and electronic devices. People who wear blue-light blocking glasses for 3 h before going to bed have higher sleep quality and better mood than people who don’t.
  *Why does blue light disrupt sleep cycles?*
4. Morphine is an effective pain medication because it mimics the body’s natural pain relief mechanism. In response to a painful stimulus, the body releases endorphins. Endorphins bind to neurons in the brain, causing dopamine to be released. Increased dopamine in the brain reduces the feeling of pain. Morphine can also bind to endorphin receptors and cause the release of dopamine. Although morphine is very effective, it is also highly addictive.
  *Why is morphine addictive?*

**Psychology**

1. Sometimes people don’t notice something that’s right in front of them. When people view rapidly presented images and are told to press a button every time they see a house, they are generally good at this. However, if two houses are presented close together, people often fail to press the button for the second house. This failure to detect the second house is called “attentional blink.”
  *Why does the attentional blink effect happen?*
2. People generally have difficulty in telling two faces apart if they come from a race other than their own. For example, in the United States, white people are typically better at recognizing white faces than black faces. The finding that it is more difficult in general to recognize faces of races different from one’s own is known as “the other-race effect.”
  *Why does the other-race effect occur?*
3. People’s word-association abilities change depending on how long it takes them to say the first word that comes to their mind. Words that are said very quickly after hearing the target word are likely to sound similar to the target. However, words that are said after a short delay are more likely to have a similar meaning to the target.
  *Why does timing affect the type of words people associate with a target?*
4. Babies are capable of doing simple arithmetic. For example, babies see a doll placed on a stage that is then hidden behind a screen. Then, they see a hand place another doll behind the screen. They look longer if the screen is dropped to reveal only one doll instead of two. This looking time difference between one and two dolls shows that babies can calculate 1 + 1 = 2.
  *Why does this looking time difference show that babies can do arithmetic?*

**Social science**

1. Men and women generally have equal intellectual abilities when it comes to learning about science and learning about the humanities. But in secondary school, boys tend to get higher grades in science and math classes, while girls tend to get higher grades in language and literature classes.
  *Why do boys and girls perform at different levels in different subjects?*
2. There are many different political systems in the world. Even among democracies, there are different ways that governments are organized. This is especially true when it comes to the number of active political parties. Some democratic countries have three or six or even ten major political parties with a role in the government, but the United States only has two.
  *Why has the United States maintained a two-party system?*
3. People who are experts in a certain field know a great deal about that field. Such people should be able to make good decisions about issues concerning that field. But when these abilities are put to the test, it is often the case that groups of ordinary, independent people make better decisions about certain issues than individual experts. This is known as “the wisdom of crowds.”
  *Why do groups make better decisions than individuals?*
4. Neighborhoods with visible petty crime, such as vandalism and graffiti, tend to also have higher levels of major crime, such as robbery and murder. When steps are taken to prevent petty crimes, the rate of major crimes goes down as well, even if nothing is done to specifically prevent major crimes. This has been called the “broken windows” theory of crime.
  *Why does cleaning up vandalism and graffiti reduce rates of major crime?*

**Questions and response options**

All Methods question: Which of the following methods could help scientists answer the italicized question?

Best Method question (displayed only if the participant selected multiple methods in response to the All Methods question): Which method would be the best way to answer the italicized question?

- Analyzing tissue samples
- Analyzing chemical composition
- Analyzing human behavior
- Analyzing atomic structure
- Analyzing society-level statistics
- Analyzing neural activity

## Footnotes

1 Biology is a complex and multi-faceted discipline, and we acknowledge that this method does not represent all areas in the field. We chose this phrasing so that non-experts would understand that this method could provide information about the biological level of the scientific hierarchy.

2 This analysis used a logistic regression predicting the proportion of trials where a participant selected a particular method from a predictor variable coding whether that method was horizontal or reductive. The regression included a random effect of subject on the slope of the predictor variable to account for repeated trials per subject. *B* = 0.40, SE = 0.07, *z* = 5.65, *p* < .001.

3 This analysis was performed in the same way as the prior analysis. The predictor variable coded whether the method was at level −1, −2, or −3, and was contrast coded to test level −1 vs. level −2 and level −2 vs. level −3. The models included random effects of subject on the intercept; more complex random-effects structures failed to converge.
